# Dose Rate Effects on the Selective Radiosensitization of Prostate Cells by GRPR-Targeted Gold Nanoparticles

**DOI:** 10.3390/ijms23095279

**Published:** 2022-05-09

**Authors:** Ana Marques, Ana Belchior, Francisco Silva, Fernanda Marques, Maria Paula Cabral Campello, Teresa Pinheiro, Pedro Santos, Luis Santos, António P. A. Matos, António Paulo

**Affiliations:** 1 Departamento de Física, Instituto Superior Técnico, Universidade de Lisboa, Avenida Rovisco Pais, 1049-001 Lisbon, Portugal; ana.sofia.97.m@gmail.com; 2 Centro de Ciências e Tecnologias Nucleares, Instituto Superior Técnico, Universidade de Lisboa, Campus Tecnológico e Nuclear, Estrada Nacional 10, Km 139.7, 2695-066 Bobadela LRS, Portugal; fmarujo@ctn.tecnico.ulisboa.pt (F.M.); pcampelo@ctn.tecnico.ulisboa.pt (M.P.C.C.); psantos@ctn.tecnico.ulisboa.pt (P.S.); apaulo@ctn.tecnico.ulisboa.pt (A.P.); 3 Departamento de Engenharia e Ciências Nucleares, Instituto Superior Técnico, Universidade de Lisboa, Av. Rovisco Pais 1, 1049-001 Lisbon, Portugal; teresa.pinheiro@tecnico.ulisboa.pt; 4 Instituto de Bioengenharia e Biociências, Instituto Superior Técnico, Universidade de Lisboa, Av. Rovisco Pais 1, 1049-001 Lisbon, Portugal; 5 Laboratório de Metrologia, Instituto Superior Técnico, Universidade de Lisboa, Av. Rovisco Pais 1, 1049-001 Lisbon, Portugal; lsantos@ctn.tecnico.ulisboa.pt; 6 Centro de Investigação Interdisciplinar Egas Moniz, Campus Universitário, Quinta da Granja, Monte de Caparica, 2829-511 Caparica, Portugal; apamatos@gmail.com

**Keywords:** radiotherapy, radiosensitizer, gold nanoparticles, prostate cancer

## Abstract

For a while, gold nanoparticles (AuNPs) have been recognized as potential radiosensitizers in cancer radiation therapy, mainly due to their physical properties, making them appealing for medical applications. Nevertheless, the performance of AuNPs as radiosensitizers still raises important questions that need further investigation. Searching for selective prostate (PCa) radiosensitizing agents, we studied the radiosensitization capability of the target-specific AuNP-BBN in cancer versus non-cancerous prostate cells, including the evaluation of dose rate effects in comparison with non-targeted counterparts (AuNP-TDOTA). PCa cells were found to exhibit increased AuNP uptake when compared to non-tumoral ones, leading to a significant loss of cellular proliferation ability and complex DNA damage, evidenced by the occurrence of multiple micronucleus per binucleated cell, in the case of PC3 cells irradiated with 2 Gy of γ-rays, after incubation with AuNP-BBN. Remarkably, the treatment of the PC3 cells with AuNP-BBN led to a much stronger influence of the dose rate on the cellular survival upon γ-photon irradiation, as well as on their genomic instability. Overall, AuNP-BBN emerged in this study as a very promising nanotool for the efficient and selective radiosensitization of human prostate cancer PC3 cells, therefore deserving further preclinical evaluation in adequate animal models for prostate cancer radiotherapy.

## 1. Introduction

Radiotherapy is one of the major therapeutic approaches used in cancer treatment, along with chemotherapy and surgery. Current data show that around 50% of cancer patients undergo radiotherapy for the treatment of local tumors [1]. This methodology is based on the deposition of ionizing energy in tumor cells, commonly through the use of high-energy gamma rays, X-rays, or ion beams, in order to damage these cells or peripheral vasculature, thus leading to tumor reduction/elimination [2]. The success of radiotherapy relies on the selective delivery of the radiation dose to tumor cells, while providing minimum energy deposition in healthy tissues.

One way to enhance ionizing energy deposition in tumor cells is by using radiosensitizers, which ideally should selectively experience uptake by the target tumoral cells when compared to the normal cells from the surrounding healthy tissues. The concept was introduced around 30 years ago when high-Z elements were explored as a way to enhance local radiation doses, taking advantage of the high attenuation capability of high-Z materials for X-rays or gamma radiation [3,4,5,6,7]. In this regard, gold nanoparticles (AuNPs) have gained much attention in recent years due to the high concentration of high-Z gold atoms (Z = 79) in a single nanoparticle (NP), their biocompatibility and functionalization simplicity [8,9,10,11,12,13]. Briefly, the interaction of photons with Au atoms leads to the occurrence of the photoelectric effect as the main source of energy emission; however, secondary emissions in the form of photoelectrons, Auger electrons and X-rays also occur. Although these secondary emissions have lower energy, they are capable of damaging biological organelles, DNA or enzymes, and to a further extent, affect the tumor microenvironment (TME) [11,14,15].

For cancer therapy applications, NPs in general can take advantage of the so-called enhanced permeability and retention (EPR) effect, which permits a higher retention of the NPs in tumor sites with minimum diffusion to other tissues [16,17]. This high tumor retention plays a crucial role in the NPs’ performance as radiosensitizing agents, making them very promising for nanobrachytherapy strategies as well. Additionally, NPs can be endowed with cancer selectivity by conjugation with tumor-targeting molecules, such as peptides or antibodies [18,19]. This cancer selectivity can be particularly relevant in cancers with low radiation sensitivity, such as prostate cancer (PCa), where the standardized radiation dose used for treatment affects both cancer and healthy cells [20,21]. Moreover, certain cancer types, namely PCa, also tend to exhibit radioresistance upon initiation of the radiotherapy treatment, and the use of radiosensitizing agents may play a crucial role in obtaining improved therapeutic outcomes [22,23].

Like the use of radiosensitizers, the dose rate effect on biological response has a high importance in radiotherapy and radiobiology but still remains a controversial issue [24]. The dose rate effects in radiotherapy are extensively described in the literature [25,26]; however, for AuNPs’ radiosensitization, there is a lack of knowledge, and the reported results are not fully consistent. Recently, Morozov and co-workers demonstrated that the dose rate influences the radiosensitization of plasmid DNA by AuNPs of various sizes [27]. The authors verified that the radiosensitization effects obtained upon an increase in the dose rate depend on the AuNPs’ size, with increasing radiosensitization for 12 nm AuNPs, no enhancement for 15 nm AuNPs and decreasing radiosensitization for 21–26 nm AuNPs. In vitro studies involving the hydroxylation of coumarin carboxylic acid and 7 nm AuNPs showed that there is an enhanced radiosensitization effect for this model reaction at increased dose rates [28,29]. On the contrary, Currell and co-workers observed that a decrease in the dose rate during the X-ray irradiation of AuNP aqueous solutions led to an increase in reactive oxygen species (ROSs) production for 32.5 nm AuNPs [30]. 

Recently, we introduced spherical AuNPs (**AuNP-TDOTA**) stabilized with a thiolated macrocyclic derivative, TDOTA (2-[4,7-bis(carboxymethyl)-10-[2-(3-sulfanylpropanoylamino)ethyl]-1,4,7,10-tetrazacyclododec-1-yl]acetic acid), with a small gold core (ca. 4 nm) and a high colloidal stability [31,32]. Their functionalization with a thiolated bombesin (BBN) peptide led to target-specific NPs (**AuNP-BBN**) with a high affinity for the gastrin-releasing peptide receptor (GRPR) and enhanced uptake in the human prostate cancer PC3 cell line overexpressing the GRPR. The favorable uptake of **AuNP-BBN** in PC3 cells encouraged us to study their potential usefulness in the selective and effective radiosensitization of prostate cancer. By considering these studies, we also wanted to take advantage of their small gold core (ca. 4 nm). In this regard, as mentioned previously, the size of the AuNPs can influence the dose deposition in irradiated cells previously treated with the NPs. More significant dose enhancement factors (DEF) have been reported for NPs with a gold core smaller than 50 nm, as there is a higher probability of self-absorption of the secondary electrons within the core of the NP for the largest NPs [27,33,34]. 

**Figure 1:**
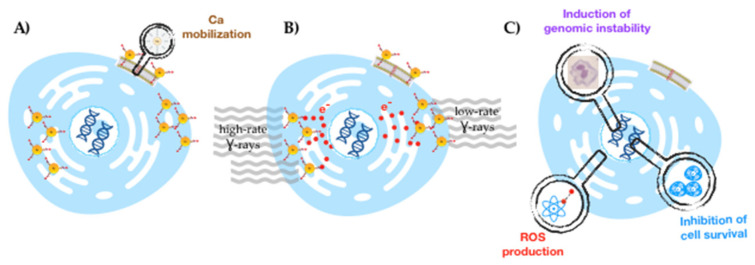
Schematic drawing of the delineated strategy for a selective radiosensitization of prostate cancer cells with target-specific AuNPs: selective GRPR-mediated cellular uptake (**A**), followed by irradiation with γ-photons (at low and high dose rates) (**B**) to obtain enhanced radiobiological effects (**C**).

Our aim in this work was to evaluate the radiosensitization effect of the target-specific **AuNP-BBN** in prostate cancer PC3 cells versus non-cancerous RWPE-1 prostate cells, in order to unravel their suitability as effective and selective PCa radiosensitizing agents (Figure 1). To this end, the study was also conducted for the non-targeted counterparts (**AuNP-TDOTA**) and encompassed the assessment of dose rate effects for selected doses of the γ-photons (Co-60 source) used in the irradiation experiments. By studying the dose rate effect, we aimed to contribute to the clarification of the complex dependence of AuNP radiosensitization on the irradiation conditions, which remains almost uninvestigated at the cellular level.

## 2. Results

### 2.1. Cytotoxicity and Cellular Uptake Studies for GRPR-Targeted and Non-Targeted AuNPs: Selection of AuNP Concentration and Irradiation Dose

**Figure 2:**
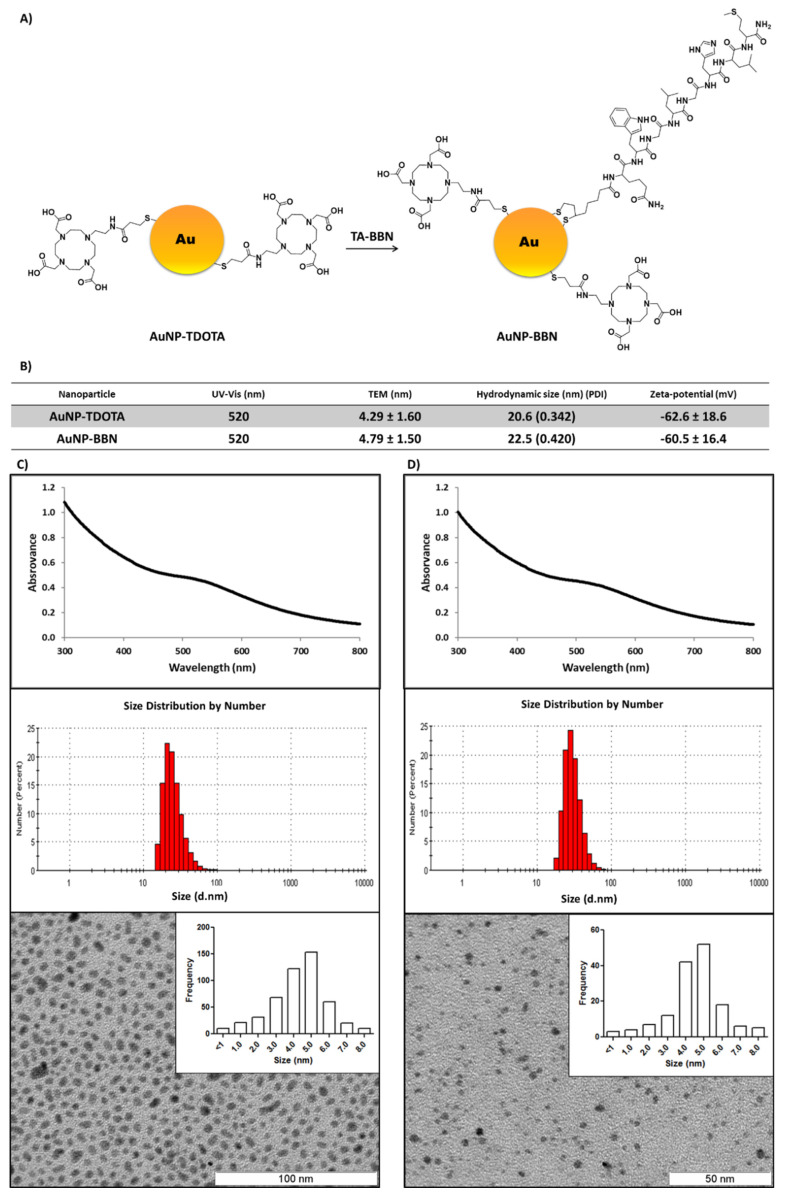
Schematic synthesis of **AuNP-BBN** from **AuNP-TDOTA** (**A**). Physico-chemical characterization of the AuNPs used in the present work (**B**). UV-Vis spectra, dynamic light scattering analyses and transmission electron microscopy imaging (with respective size histogram), of **AuNP-TDOTA** (**C**) and **AuNP-BBN** (**D**).

As mentioned above, the AuNPs evaluated in this study as radiosensitizers were stabilized with a thiolated DOTA derivative (TDOTA) and contained or did not contain a thiolated BBN derivative (TA-BBN). The detailed synthesis and characterization of both types of nanoparticles (**AuNP-TDOTA** and **AuNP-BBN**) have been described previously by us [31], and are summarized in Figure 2. Briefly, the **AuNP-TDOTA** and **AuNP-BBN** showed estimated core sizes of 4.29 ± 1.60 and 4.79 ± 1.50 nm, respectively, with hydrodynamic sizes close to 20 nm.

**Figure 3:**
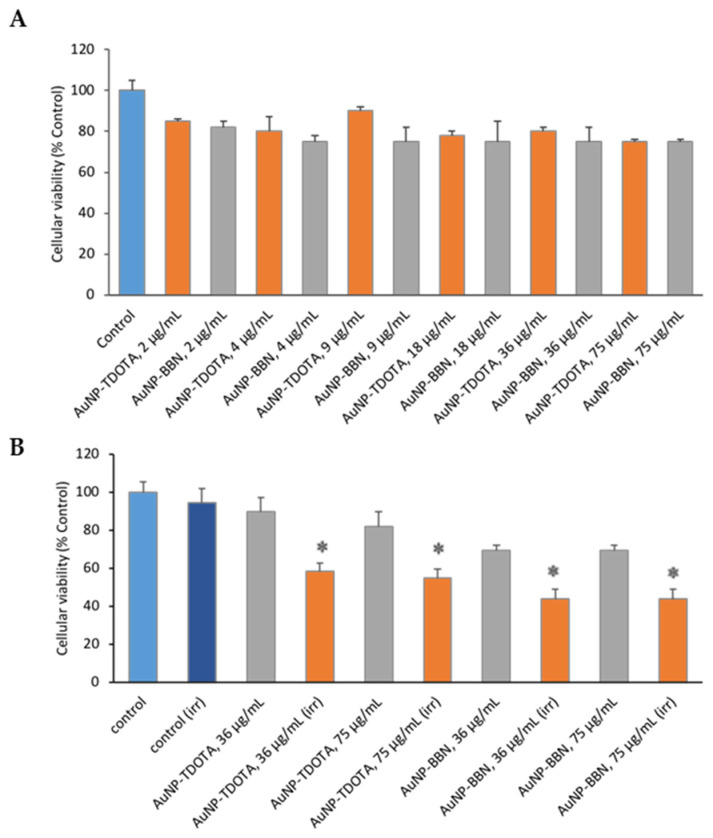
(**A**) Cellular viability of PC3 cells after 4 h of incubation with the AuNPs (**AuNP-TDOTA** and **AuNP-BBN**) at different gold concentrations (2–75 µg/mL). (**B**) Cellular viability before and after irradiation at 2 Gy for selected concentrations. The MTT assay was performed 72 h after irradiation. For comparison, the same procedure was followed for the non-irradiated cells. Statistical significance was calculated using two-tailed Student’s *t*-test with non-irradiated cells as the control (* *p* ≤ 0.05). Data are expressed as the percentage of cellular viability (mean ± SD).

Prior to the irradiation studies, we screened the cytotoxic activity of the AuNPs in PC3-cells, using the [1-(4,5-dimethylthiazol-2-yl)-2,5-diphenyl tetrazolium] (MTT) assay to assess the viability of the cells treated with increasing AuNP concentrations, in the range of 2–75 µg/mL. This study was expected to guide the selection of adequate concentrations of the AuNPs for the irradiation studies presented in this work, as the AuNPs should not exhibit intrinsic cytotoxic activity at the concentrations used for the evaluation of their radiosensitizing capabilities. As can be observed in Figure 3A, the loss of cellular viability did not exceed 75% compared to the controls over the whole range of AuNP concentrations. 

Concerning the selection of the dose value to be used in the evaluation of the AuNPs as radiosensitizers, we studied the influence of the cell irradiation on their viability by exposing PC3 cells to a range of doses (0.5 to 6 Gy) using a Co-60 source (dose rate of 1 Gy/min). Our assumption in choosing a certain dose value was that it should be high enough to induce radiosensitization and, at the same time, not so high as to compromise the cellular viability. In this study, we obtained cellular survival rates of 61%, 37% and 13% for 1, 2 and 4 Gy, respectively (Supplementary Figure S1). Based on these results, we chose to use a dose value of 2 Gy, because: (i) the inhibition of cell viability is below 50% and (ii) this dose value is common in radiotherapy treatments with dose fractionation. Thereafter, we studied the radiosensitizing capability of the AuNPs at 36 and 75 µg/mL concentrations for the selected dose value, i.e., after irradiation of PC3 cells with 2 Gy of Co-60 gamma rays, by evaluating the effect on the cellular viability using the MTT assay. The results are depicted in Figure 3B. 

Following the irradiation of cells with 2 Gy, the results show a significant loss of cellular viability for the irradiated cells when compared with their corresponding controls (Figure 3B). For the tested concentrations (36 and 75 µg/mL), and for both types of nanoparticles (**AuNP-TDOTA** and **AuNP-BBN**), the results reveal a non-statistical difference between the effects on the cellular viability, both for irradiated and non-irradiated cells, in line with the results depicted in Figure 3A. Therefore, the concentration of 36 µg Au/mL was selected for further irradiation studies, as a compromise to obtain high radiosensitizing effects with lower AuNP amounts.

The radiosensitizing potential of **AuNP-TDOTA** and **AuNP-BBN** strongly depend on their uptake by the PC3 cells. Therefore, studies were carried out to quantify the levels of Au inside the cells and to evaluate any differences between the AuNPs. At the selected AuNPs concentration, i.e., 36 µg Au/mL, we studied the cellular uptake of **AuNP-TDOTA** and **AuNP-BBN** in PC3 and the non-tumoral RWPE-1 cells by measuring the Au content of the cells treated with the AuNPs, using particle-Induced X-ray emission (PIXE). The results are presented in Table 1. As can be seen, the cellular uptake, for both AuNPs, was higher in tumoral cells (PC3 cell line) when compared with non-tumoral ones (RWPE-1 cell line). However, this difference was more striking for **AuNP-BBN** (ca. 4.5-fold increase) than for **AuNP-TDOTA** (ca. 1.4-fold increase). Remarkably, **AuNP-BBN** has a much higher cellular uptake, being more pronounced in PC3 cells due to its specificity towards the GRPR overexpressed in prostate cancer cells, which is in accordance with previous studies reported for these AuNP platforms [24]. 

As PIXE is a multielemental analytical technique, the concentrations of the cellular physiological elements could also be measured. In relation to calcium levels, the incubation of **AuNP-BBN** induced a 4-fold increase in cellular Ca^2+^ compared to **AuNP-TDOTA** in the PC3 cells (324 ± 4 vs. 74 ± 2 in µg/g wet weight). The PC3 cells treated with **AuNP-BBN** also showed a 10-fold increase in the cellular Ca relative to controls (controls, 36 ± 2 in µg/g wet weight). This effect was not observed with the non-tumoral RWPE-1 cells, with similar Ca^2+^ levels upon treatment with both types of AuNPs (41 ± 3 vs. 53 ± 1 in µg/g wet weight). 

### 2.2. Irradiation Studies: Tumoral versus Non-Tumoral Cells and Dose Rate Effect

Based on the promising cellular uptake results, we proceeded with a more detailed evaluation of the cellular damage induced in PC3 and RWPE-1 cells after incubation with **AuNP-TDOTA** and **AuNP-BBN** followed by exposure to γ-radiation from Co-60. 

#### 2.2.1. Cellular Survival—Colony Formation

**Figure 4:**
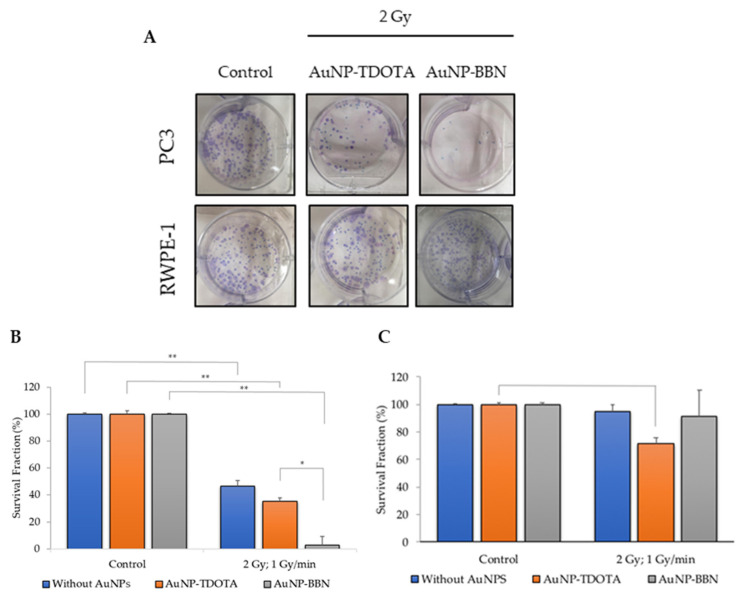
Inhibitory effect of **AuNP-TDOTA** and **AuNP-BBN** at a concentration of 36 µg Au/mL followed by 2 Gy of *γ*-radiation (dose rate of 1 Gy/min) on PC3 and RWPE-1 cells’ proliferation evaluated by the clonogenic assay, in comparison with non-irradiated controls. (**A**) Representative images, (**B**) quantification of the survival fractions on PC3 cells, and (**C**) quantification of the survival fractions on RWPE-1 cells. Statistical significance was calculated using two-tailed Student’s *t*-test (** *p* ≤ 0.001) (* *p* ≤ 0.05). The results were calculated from independent biological replicates (*n* = 2) and are given as the mean ± S.E.M.

Taking into consideration a potential increase in chromosomal damage and genomic instability in cells incubated with **AuNP-BBN** and irradiated with γ-photons, we next investigated if this would lead to an enhanced inhibition of PC3 cell proliferation, based on the clonogenic assay and in comparison with **AuNP-TDOTA**. The clonogenic assay evaluates the ability of a single cell to grow into a colony, i.e., to undergo continuous proliferation, and is often used to study the effect of ionizing radiation on the survival of cancer cells, either for external beam radiation therapy (EBRT) or for TRT with medical radionuclides [35]. The results of the clonogenic assays for PC3 and RWPE-1 cell lines, expressed as the survival fraction, are presented in Figure 4B,C, respectively. 

From Figure 4B, we can see that there was a significant loss in cellular proliferation ability for PC3 cells exposed to 2 Gy of γ-radiation after incubation with both AuNPs. The observed survival fractions were 46.6 ± 1.2%, 35.2 ± 7.8% and 2.7 ± 1.78 for irradiation only or for irradiation combined with **AuNP-TDOTA** and **AuNP-BBN**, respectively. Contrastingly, RWPE-1 cells showed a less evident loss of cellular proliferation capacity, as shown in Figure 4C. The observed survival fractions were 95.0 ± 4.9%, 71.3 ± 4.7% and 91.3 ± 18.9%, respectively, for the same irradiation conditions used in the experiments with the PC3 cells. 

#### 2.2.2. Induction of Genomic Instability

**Figure 5:**
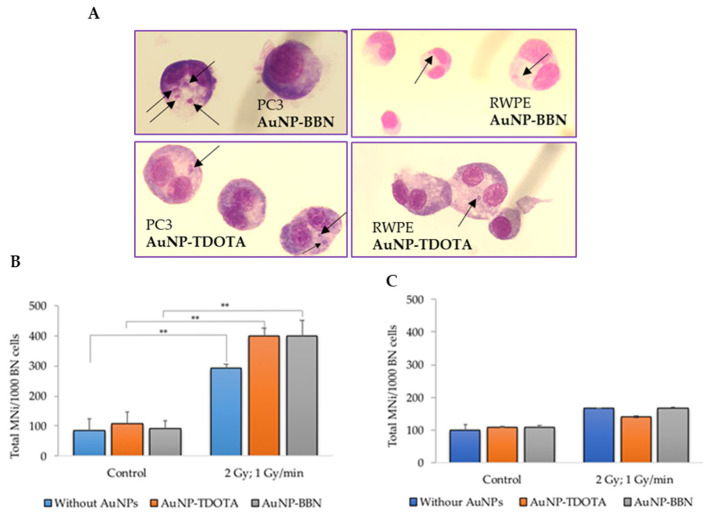
Genotoxic effects, in PC3 and RWPE-1 cells, after incubation with **AuNP-TDOTA** and **AuNP-BBN** at a concentration of 36 µg Au/mL followed by 2 Gy of *γ*-radiation (dose rate of 1 Gy/min), evaluated by the CBMN assay. (**A**) Representative light microscopy images of DNA damage in the tumoral cells PC3 and non-tumoral cells RWPE-1, respectively. The arrows indicate the presence of MNi. Giemsa staining was used to visualize the nuclei and cytoplasm under 40× magnification. (**B**,**C**) Average number of MNi per 1000 BN cells in PC3 cells and RWPE-1 cells, respectively. Statistical significance was calculated using two-tailed Student’s *t*-test with irradiation-only cells as the control (** *p* ≤ 0.001) The results were calculated from independent biological replicates (*n* = 2) and are given as the mean ± S.E.M.

We next investigated whether the cell survival and cellular uptake results were correlated with the induction of genomic instability, using the cytokinesis-blocked micronucleus (CBMN) assay, as described in the literature [36]. Micronuclei (MNi) are extranuclear bodies that form during anaphase, as a result of unrepaired chromosome breaks, DNA misrepair or formation of acentric chromosome fragments, namely upon cell exposure to ionizing radiation. The cells were exposed to 2 Gy of γ-radiation (Co-60, with a dose rate of 1 Gy/min) after incubation with both compounds, **AuNP-TDOTA** and **AuNP-BBN**. The results are expressed as the total number of MNi per 1000 binucleated (BN) cells (Figure 5B,C).

**Figure 6:**
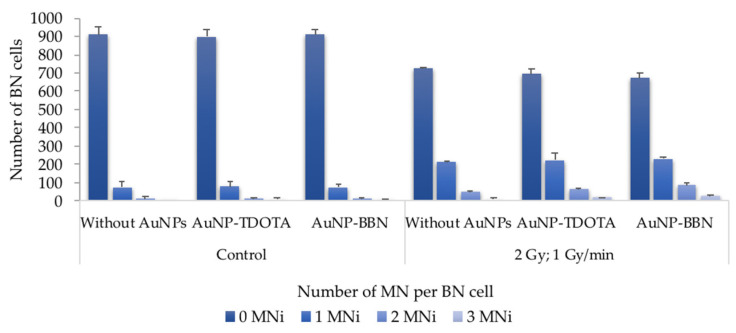
Average distribution of MNi per 1000 BN cells, 0, 1, 2 and 3 MNi, respectively, in PC3 cells irradiated with 2 Gy, following incubation with **AuNP-TDOTA** and **AuNP-BBN** at a concentration of 36 µg Au/mL and in PC3 control cells. The results were calculated from independent biological replicates (*n* = 2) and are given as the mean ± S.E.M.

For the PC3 cell line, the results reveal a significant increase in the total number of MNi in irradiated cells when compared with non-irradiated cells, incubated or not with the AuNPs (Figure 5B). Such an increase did not occur in the RWPE-1 cells (Figure 5C). Of note is the fact that no statistical difference in the MNi number was observed in PC3 cells treated with **AuNP-TDOTA** or **AuNP-BBN** and irradiated with γ-photons. However, observing the distribution of the MNi per 1000 BN cells (Figure 6), we noticed complex DNA damage, evidenced by the occurrence of multiple MNi per BN cell in the case of PC3 cells irradiated with a 2 Gy dose, after exposure to **AuNP-BBN**. 

#### 2.2.3. Generation of Reactive Oxygen Species (ROSs)

In combination with IR, the contribution of AuNPs for an increased radiosensitization of tumor cells can occur through enhanced ROS production, as previously reported by others [23]. Taking into consideration that ROSs can play an important role in indirect radiation-induced DNA damage, we evaluated the production of ROSs, comprising mainly peroxides hydrogen peroxide (H_2_O_2_) and hydroxyl radical (HO^•^), using the cell-permeant indicator H2DCF-DA and the superoxide (O2^•–^) using the NBT assay after irradiation of PC3 cells incubated with **AuNP-TDOTA** and **AuNP-BBN**. 

**Figure 7:**
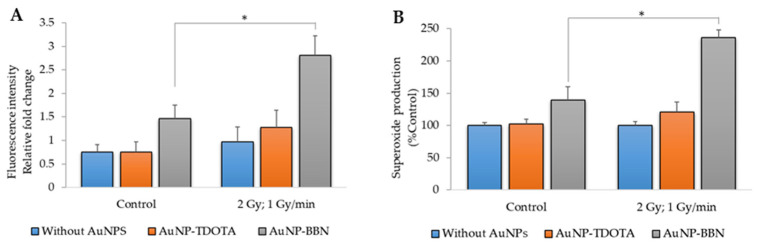
ROS production in PC3 cells after irradiation with 2 Gy of *γ*-radiation (dose rate of 1 Gy/min) and incubation with AuNP-TDOTA and AuNP-BBN at a concentration of 36 µg Au/mL: (**A**) peroxides (H_2_O_2_ and HO^•^) and (**B**) superoxide (O_2_^•–^). Statistical significance was calculated using two-tailed Student’s *t*-test with irradiation-only cells as the control (* *p* ≤ 0.05) The results were calculated from independent biological replicates and are given as the mean ± S.E.M.

As shown in Figure 7, the incubation of control (non-irradiated) PC3 cells with **AuNP-BBN** was accompanied by an increase in the basal ROS level. However, this did not translate into significant cytotoxicity for these AuNPs, according to the cell viability studies described above. Notably, γ-irradiation of PC3 cells incubated with both **AuNP-TDOTA** and **AuNP-BBN** led to an increase in ROS levels, compared to the respective non-irradiated control cells, but again the ROS levels were higher for cells previously treated with **AuNP-BBN**. 

#### 2.2.4. Dose Rate Effect

The impact of IR on cells depends significantly on the particle fluence of radiation per unit of time, the so-called dose rate effect [25,26]. Having this in mind, we have investigated whether the AuNPs’ radiosensitization could be dependent on the dose rate effect. Due to the modest radiosensitization effects observed for the RWPE-1 cell line, this dose rate effect study was only conducted for the PC3 prostate cancer cell line. 

**Figure 8:**
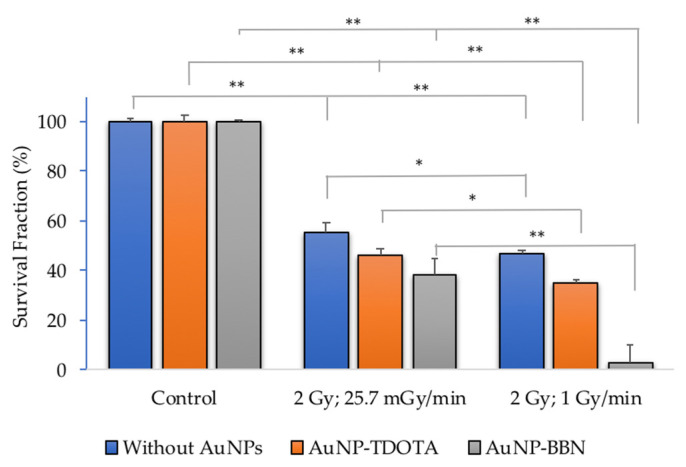
Quantification of the survival fractions in PC3 cells irradiated with 2 Gy, using two different dose rate values (25.7 mGy/min and 1 Gy/min) of Co-60, following incubation with **AuNP-TDOTA** and **AuNP-BBN** at a concentration of 36 µg Au/mL. Statistical significance was calculated using two-tailed Student’s *t*-test with irradiation-only cells as the control (* *p* ≤ 0.05) and between treatment groups (** *p* ≤ 0.001). The results were calculated from independent biological replicates (*n* = 2) and are given as the mean ± S.E.M.

First, to evaluate if the dose rate effect could affect the radiosensitization capability of the AuNPs, we studied the cellular proliferation rate of PC3 cells treated with **AuNP-TDOTA** and **AuNP-BBN** and then exposed to a 2 Gy radiation dose using two different dose rates: (25.7 mGy/min and 1 Gy/min) (Figure 8 and Table S2). 

The results from the clonogenic assay showed a loss of cellular proliferation ability using both dose rate values when the irradiated cells compared with their corresponding controls. Concerning the effect of the dose rate value on the survival fractions observed for each treatment group, we can conclude that a higher dose rate induces higher AuNP radiosensitization. As described in the SI, the amplification factor (AF) values were calculated for the radiation-induced cell death efficiency in cells incubated with AuNPs (**AuNP-TDOTA** or **AuNP-BBN**) compared to irradiation alone experiments (i.e., without AuNPs) (Figure S3). It is clearly evident that the highest amplification of cellular death was observed for **AuNP-BBN** at a 1 mGy/min dose rate. 

**Figure 9:**
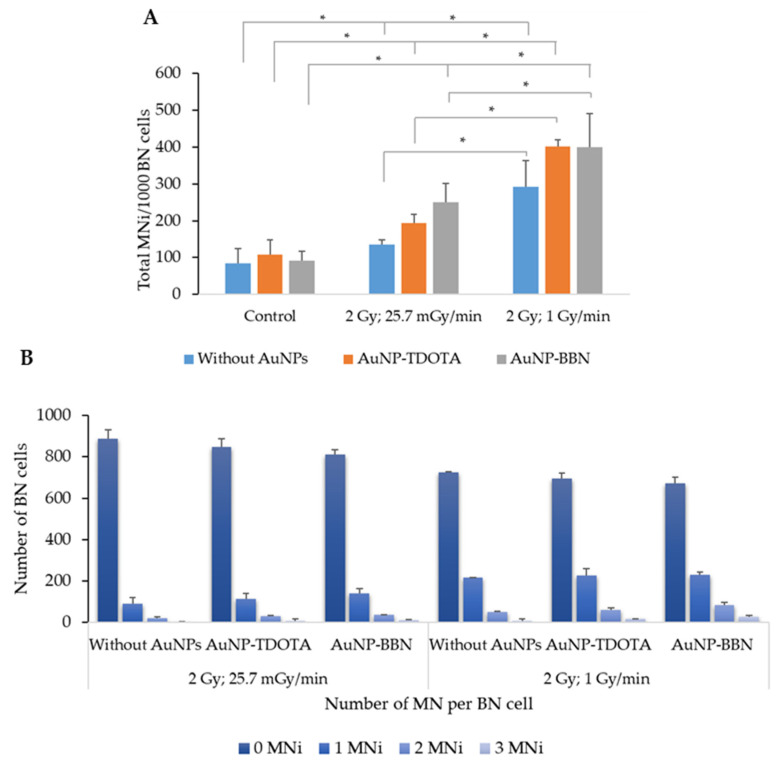
Genotoxic effects in PC3 cells evaluated by the CBMN assay, after incubation of the cells with AuNP-TDOTA and AuNP-BBN at a concentration of 36 µg Au/mL followed by irradiation with 2 Gy of Co-60 radiation. (**A**) Average number of MNi per 1000 BN cells, in PC3 cells. (**B**) Average distribution of MN per 1000 BN cells. The results were calculated from independent biological replicates (*n* = 2) and are given as the mean ± S.E.M, (* *p* ≤ 0.05).

As before, we also studied the effect of the dose rate on the genotoxic effects induced by the treatment of PC3 cells with **AuNP-TDOTA** and **AuNP-BBN**, followed by irradiation with γ-photons. The results reveal an increase in the total number of micronuclei (MNi) on the irradiated cells when compared with its corresponding non-irradiated controls. In addition, the cells exposed to a lower dose rate (25.7 mGy/min) exhibited decreased cellular damage expressed by: (i) a lower number of MNi/1000 BN cells (Figure 9A) and (ii) a lower number of cells exhibiting multiple MNi (Figure 9B). 

### 2.3. Cellular Morphological Alterations after Irradiation

**Figure 10:**
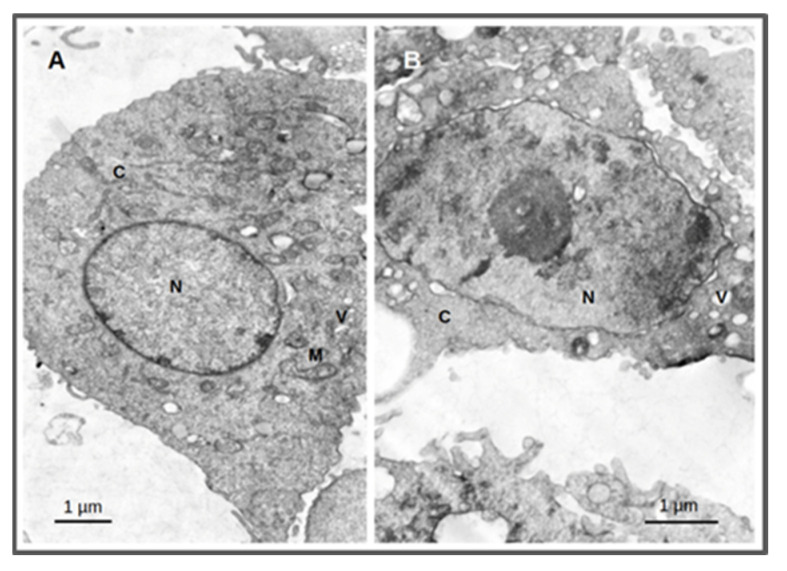
Thin-section transmission electron microscopy images of PC3 cells treated for 4 h with **AuNP-BBN** at a concentration of 36 µg Au/mL followed by irradiation with 2 Gy (1 Gy/min). Samples were analyzed and photographed in a JEOL 1200-EX electron microscope. Irradiated PC3 cells (**A**); irradiated PC3 cells after incubation with **AuNP-BBN.** (**B**); N = nucleus; C = cytoplasm; M = mitochondria; V = vacuoles. Bars = 1 micrometer.

The cellular effects after irradiation were evaluated by transmission electron microscopy (TEM) using the PC3 cells. This technique enables the visualization of the structural alterations of the cells in the nanometer range. Cellular damage was assessed by incubating the cells with **AuNP-BBN** at a concentration of 36 µg Au/mL followed by irradiation with 2 Gy (1 Gy/min) as described above. Untreated PC3 cells (control) were also included using the same irradiation conditions. Electron microscopic study of irradiated PC3 cells treated with **AuNP-BBN** showed disruption of nuclear chromatin, reduction in cytoplasm and vacuolization of cytoplasmic organelles, while the irradiated control PC3 cells showed normal cellular ultrastructure, intact nuclei and normal cell organelles (Figure 10).

## 3. Discussion

Our main goal was to promote selective and enhanced radiosensitization of prostate cancers cells based on GRPR-targeted AuNPs. For this purpose, the tested **AuNP-BBN** needed to show an intrinsically low cytotoxicity in order to avoid systemic cytotoxic effects from their side. On the other hand, they needed to present an enhanced uptake in the targeted prostate cancer PC3 cells, with a reduced uptake in neighboring normal cells. The performed cytotoxicity studies showed that **AuNP-BBN**, like the non-targeted congeners **AuNP-TDOTA**, has a rather low cytotoxicity against the PC3 cell line (Figure 3A), up to the maximum evaluated concentration (75 μg Au/mL), which is an important requisite for their potential application as radiosensitizers for prostate cancer treatment. Most relevantly, **AuNP-BBN** showed a much higher uptake in the PC3 cells when compared with normal RWPE-1 prostate cells, with a ca. five-fold increase in uptake in the tumoral cells. By contrast, **AuNP-TDOTA** showed a relatively similar uptake in both cell lines, with a much lower uptake (ca. 15 times less) in the PC3 cell line when compared to **AuNP-BBN** (Table 1). The cellular uptake results corroborate the importance of the attachment of the bombesin derivative at the AuNP surface to promote a specific uptake by the target tumor cells. 

AuNPs once bound to the plasma membrane are thought to be carried inside the cells mainly via the endocytosis pathway. This process can induce membrane depolarization, and this can result in an increase in Ca concentration inside the cells. The observed increase in intracellular Ca concentrations associated with the enhancement of targeting accumulation by **AuNP-BBN** cellular uptake suggest that these Au nanoparticles may influence membrane permeability, depolarization and the mobilization of Ca into the PC3 cells [37]. Previously, we have shown that **AuNP-BBN** has a high affinity (IC_50_ = 0.045 ± 0.003 µg Au/mL) towards the GRPR. Their ability to promote Ca^2+^ mobilization in the PC3 cells seems to indicate that they have an agonist character, as it has been reported that BBN agonists can induce rapid calcium mobilization in GRPR-positive cell lines, namely in PC3 cells [38,39]. 

The cell survival of PC3 cells treated with **AuNP-BBN** and, thereafter, irradiated with a 2 Gy radiation dose, was negligible (<5%) and much lower than the survival of the cells irradiated with the same dose that were not exposed to the AuNPs. In the case of the RWPE-1 cell line, such a difference was not so striking, being almost non-existent (Figure 4B). For **AuNP-TDOTA**, a much lower reduction in the survival fraction was observed for PC3 cells treated with these non-targeted AuNPs and irradiated with γ-photons, though a reasonable cell survival was still observed (>20%). Interestingly, **AuNP-TDOTA** caused a stronger reduction in the survival fraction of post-irradiated RWPE-1 cells than **AuNP-BBN**. These findings are consistent with the cellular uptake results, highlighting that **AuNP-BBN** acts as a more efficient and selective radiosensitizer of PC3 cells when compared with **AuNP-TDOTA**.

The clonogenic survival fraction is a macroscopic outcome in radiobiology experiments that depends on the proliferation rate of the cells and their ability to form colonies, which are influenced by diverse biological processes such as genomic instability or oxidative stress. Thus, we studied the formation of micronuclei (Mni) and ROS production in PC3 and RWPE-1 cells treated with **AuNP-TDOTA** and **AuNP-BBN**, and irradiated with a 2 Gy radiation doses, as a measure of genomic instability and oxidative stress processes, respectively. Concerning the Mni formation, the differences between the cells treated with **AuNP-TDOTA** and **AuNP-BBN**, either for PC3 or RWPE-1 cell lines, were negligible (Figure 4). However, apparently, the exposure of PC3 cells to **AuNP-BBN** led to the formation of more complex DNA damage in PC3 cells, as evidenced by the more intense occurrence of multiple Mni per BN cell in the case of **AuNP-BBN** (Figure 6). Consistently with this DNA damage, TEM images of irradiated PC3 cells treated with **AuNP-BBN** showed an impressive nuclear chromatin disruption (Figure 10), among other cellular ultrastructural alterations, which was absent in irradiated PC3 cells. 

We observed a clear enhancement in ROS production in irradiated PC3 cells treated with **AuNP-BBN** when compared with those treated with **AuNP-TDOTA**, for both the peroxide and superoxide species. This trend correlates well with the diminished survival rate that was observed for the cells exposed to the **AuNP-BBN** nanoparticles. These results corroborate the excellent radiosensitizing capabilities of **AuNP-BBN**, which might eventually result from the combination of being a GRPR agonist promoting Ca^2+^ mobilization and the interaction of the gold atoms with the incident γ-photons. In fact, the free Ca^2+^ ion is a mediator in multiple processes, from mitochondrial function and gene transcription to apoptosis [40,41,42]. Therefore, the increase in intracellular Ca concentrations associated with an expressive uptake of **AuNP-BBN** is in line with the observed genomic instability and increased levels of ROSs in PC3 cells after gamma radiation exposure. Currently, there is a large body of evidence contenting that both Ca^2+^ and ROS are mediators of cell death [40]. Additionally, imbalances of the redox status in cells are intimately related to the activation of intracellular Ca channels [43]. In the particular case of cancer cells, the Ca^2+^-ROS relationship stems from the ability of Ca^2+^ to regulate the cellular redox environment. In addition, the subcellular location of Ca^2+^ stores and the sites of ROS production, especially the ER-mitochondrial interface and the plasma membrane, are intimately linked, evidencing the intervention of these mediators in diverse cellular processes [43].

Lastly, we have dose rate effects on the γ-irradiation of PC3 cells previously treated with **AuNP-TDOTA** and **AuNP-BBN**. Several authors have studied the dose rate effects on the irradiation of tumor cells, but not those treated with AuNPs. The reported results are discrepant in terms of showing induced genetic damage and cell death outcomes, which are dependent on the studied cell lines. Some authors verified that lower dose rates induce more micronuclei and apoptosis in several cancer cell lines compared to higher dose rates, with decreased cell survival in some cases [44,45,46]. Others have shown that low dose rates are less deleterious to the survival of irradiated cells, while a few studies have reported no differences in the survival of several cancer cells treated with different dose rates [47,48]. In our study, in the absence of the AuNPs, the use of two different dose rates (25.7 mGy/min vs. 1 Gy/min) did not lead to largely different survival fractions for the irradiated PC3 cells either, although the largest dose rate seemed to induce more pronounced genomic instability. A relatively similar scenario was observed for the PC3 cells treated with **AuNP-TDOTA**, which concerns the influence of the dose rate on the radiobiological effects resulting from their γ-irradiation. 

Remarkably, the treatment of the cells with **AuNP-BBN** led to a much stronger influence of the dose rate on the survival of the irradiated PC3 cells, as well as on their genomic instability. In fact, the use of the highest dose rate of 1 Gy/min lead to a much lower survival fraction (<5%) and more complex genomic damage, as evidenced by an increased number of multiple Mni per BN cell (Figure 8 and Figure 9). Thus, these results show that the flux of photons hitting the PC3 cells has a strong influence on the radiosensitizing effects exerted by the **AuNP-BBN** nanoparticles, undergoing a high specific uptake by these tumoral cells. Most probably, the irradiation at lower dose rates leads to the emission of a minor number of electrons (e.g., photoelectrons or Auger electrons) per unit of time upon excitation of the Au atoms and, ultimately, to less ROS production. Under these circumstances, it is more probable that DNA damage repair has a better ability to compete with production of lethal DNA lesions, therefore leading to reduced cell killing. Besides these physical effects, chemical and biological factors should also be considered to explain the enhanced radiosensitizing capabilities exhibited by **AuNP-BBN**. For instance, as discussed above, we have shown that **AuNP-BBN** promotes the Ca^2+^ mobilization in PC3 cells, and there is a Ca^2+^-ROS relationship that reflects the ability of Ca^2+^ to regulate the cellular redox environment. On the other side, metallic NPs can inhibit antioxidant enzymes such as thioredoxin reductase (TrxR), as reported by other authors [49,50,51,52]. Thus, the AuNPs may be serving a dual function by increasing ROS production as well as inhibiting the enzymatic systems responsible for the reduction in oxidative stress in the cells, and consequently leading to cell death. All in all, this hypothesis is very well-fitted to our results since a greater radiosensitizing effect was expected when the dose rate was decreased due to the weakening of the antioxidant defense system.

In summary, **AuNP-BBN** emerged in this study as a very promising nanotool for the efficient and selective radiosensitization of human prostate cancer PC3 cells and are therefore deserving of further preclinical evaluation in adequate animal models for external beam radiation therapy (EBRT) of prostate cancer. For these future preclinical animal studies, the use of **AuNP-BBN** offers the possibility of exploring systemic or intratumoral administration routes. The intratumoral administration will allow us to explore a kind of nanobrachytherapy approach that might offer further selectivity in the radiosensitization of prostate cancer by **AuNP-BBN**. 

## 4. Materials and Methods

### 4.1. Synthesis and Dissolution of AuNPs

The nanoparticles **AuNP-TDOTA** and **AuNP-BBN** were synthesized as described elsewhere [31]. For the cellular studies, the AuNPs were dissolved in distilled water and diluted in cell culture medium to the desired final concentrations, under sonication to obtain solutions with a homogenous dispersion of the nanoparticles. 

### 4.2. Irradiation Experiments

Cell irradiation was carried out by delivering 2 Gy of γ-rays using two Co-60 irradiators: PRECISA-22 (Graviner Manufacturing Company, Ltd., Buckinghamshire, UK) and ELDORADO 6 (AECL Medical Products, Chalk River, Ontario, Canada) [53]. Briefly, Precisa-22 contains four sources placed inside stainless steel cylinders, where they move according to the action of a pneumatic system. The samples were placed on a support that rotates automatically to achieve a dose rate of 1 Gy/min, aiming at increasing the dose rate uniformity. The dosimetric system used to obtain the average dose rate in the wells of the cell plates was the ionization chamber FC65P. The deviations from the wanted value of 1 Gy/min were very small, and the overall variation in the dose rate in the plates was below 10%. 

ELDORADO 6 was installed in the Metrology Laboratory of Ionising Radiation at CTN. At a distance of 1 m, with this irradiator, the dose rate was 25.7 mGy/min. The uniformity of the dose rate was guaranteed for the entire cell plate (10 cm × 10 cm).

### 4.3. Cell Culture

PC3 human prostate cancer cells (ECACC, England, UK) were grown in RPMI, and the non-tumoral cells, RWPE-1, were grown in keratinocyte growth medium (KSFM) supplemented with 0.05 mg/mL bovine pituitary extract and 5 ng/mL recombinant human epidermal growth factor. Both culture media were further supplemented with 10% heat-inactivated fetal bovine serum (FBS) and 1% penicillin–streptomycin. Cells were grown at 37 °C in a humidified atmosphere of 5% CO_2_.

### 4.4. MTT Assay

Cells were seeded in 96-well culture plates at a density of 2 × 10^4^ cells/well and left to adhere overnight at 37 °C. Cells were then incubated with the desired AuNPs for 4 h at 37 °C. Thereafter, the cells were irradiated with 2Gy of γ-radiation. Then, the medium containing the NPs was replaced by fresh culture medium, and the cells were maintained at 37 °C for 72 h. After this time, the cells were washed with PBS and then incubated with MTT (0.5 mg/mL PBS) for 3 h at 37 °C. The MTT solution was then removed, and the formed insoluble blue formazan crystals were dissolved in DMSO. The absorbance of this colored solution was measured at 570 nm in a plate spectrophotometer (Power Wave Xs; Bio-Tek). Each test was performed with at least 4 replicates. Control experiments were performed for non-irradiated cells, without AuNPs or incubated previously with **AuNP-TDOTA** and **AuNP-BBN**. The results are expressed as the percentage of the viable cells in relation to the control (untreated cells).

### 4.5. Cellular Uptake—PIXE

The concentrations of Au in PC3 and RWPE-1 cells were determined by particle-induced X-ray emission (PIXE) technique, using the Van de Graaff accelerator of Instituto Superior Técnico. Both PC3 and RWPE-1 cells were incubated with **AuNP-TDOTA** and **AuNP-BBN** at 36 µg Au/mL for 4 h. The cell pellets were obtained by centrifugation after washing the cells with PBS to remove the medium, freeze-dried and microwave-assisted acid digestion (350 W, 15 s) using nitric and hydrochloric acids (1:3 molar ratio) together with yttrium (Y) (100 mg/L) as an internal standard. The detailed methodology encompassing sample preparation, PIXE analysis, concentration calculation and quality control was previously described [54,55]. The elemental concentrations of Au were obtained in µg/g dry weight and converted to wet weight or ng/10^6^ cells as appropriate. 

### 4.6. Cellular Proliferation and Colony Formation Assay

The cells were incubated with the NPs approximately 2 h before the irradiation. Immediately after irradiation, they were transferred to 6-well plates to make it easier to discriminate between the colonies after they formed. They were then left to grow for 13 days in the incubator. After this time, the culture medium was removed from the wells and the cells were fixed with an ice-cold solution of methanol and acetic acid (3:1) for 20 min. Then, the colonies were stained with a crystal violet solution for 7 min, rinsed with tap water and left to dry at room temperature, as previously described [56]. Subsequently, the number of colonies was counted in all of the plates and the survival fraction for each dose was calculated. In this work, a collection of cells was considered a colony when there were more than 50 cells in the group.

### 4.7. Cytokinesis-Blocked Micronucleus (CBMN) Assay

PC3 and RWPE-1 cells were seeded at a density of 1 × 10^5^ cells/well in a 6-well culture plates and allowed to attach and grow for 24 h at 37 °C. Cells were then incubated with the **AuNPDOTA** and **AuNP-BBN** for 2 h at 37 °C. After, cells were irradiated with 2 Gy using both PRECISA and ELDORADO. Immediately after irradiation, the medium containing the NPs was replaced by fresh culture medium and the cells maintained at 37 °C. Cytochalasin B (2 µg/mL) was added 22 h after exposure to radiation, and the cells were incubated for an additional 24 h. Then, cells were fixed and stained, as previously described [57]. For each experiment, 1000 binucleated cells (BN) were scored, and two independent experiments were performed for each cell line and irradiation conditions.

### 4.8. ROS Production

#### 4.8.1. Superoxide Radical Production (NBT Assay)

The NBT assay (NBT = 2,2′-bis(4-Nitrophenyl)-5,5′-diphenyl-3,3′-(3,3′-dimethoxy-4,4′-diphenylene)ditetrazolium chloride) was adapted from a previously described method, using a 96-well plate format [58]. NBT is an artificial electron acceptor. Upon interaction with superoxide, the tetrazole ring is disrupted and the tetrazoinyl radical generated, which subsequently underwent dismutation to generate purple-blue water-insoluble formazan crystals [59]. Briefly, after incubation of the PC3 cells (2 × 10^4^/well) with the AuNPs for 4 h at 37 °C, the cells were irradiated at 2 Gy (dose rate of 1 Gy/min) for 2 min. Then, 20 μL of a 10 mg/mL NBT solution in water was added to the cellular medium, and incubation was subsequently carried out for 1 h at 37 °C. The culture medium was then discarded, and the blue formazan particles were dissolved in 200 μL of 90% DMSO (90% DMSO:10% NaOH 0.1 N with 0.1%SDS). NBT formazan was measured at 560 nm using a plate spectrophotometer. Results (mean ± SD) are expressed as % of controls (untreated cells).

#### 4.8.2. Peroxide Production

The production of ROS (peroxides) after γ irradiation was measured by using the fluorescent probe H_2_DCF-DA (dihydro-2′7’dichlorofluorescein diacetate), as previously described [60]. PC3 cells (2 × 10^4^/well) were seeded in 96-well plates and left to grow overnight. Then, the medium was replaced with a solution of 10 μM H_2_DCF-DA in colorless DMEM/F12, and cells were incubated at 37 °C for 30 min. This solution was then removed, and cells were incubated with the AuNPs solutions in colorless DMEM/F12 at 36 µg Au/mL for 1 h and then were irradiated at 2 Gy (Dose rate of 1 Gy/min) for 2 min. Dichlorofluorescein (DCF) fluorescence was measured using at 492 nm excitation and 517 nm emission using a Varioskan LUX scanning multimode reader (ThermoFisher Scientific). Results (mean ± SD) are expressed as relative luminescent units (RLU).

#### 4.8.3. Cellular Morphological Alterations by TEM

PC3 cells seeded into 6-well plates at approx. 70% confluence (ca. 10^6^ cells) were treated with **AuNP-BBN** at a concentration of 36 µg Au/mL. After 4 h of incubation, treated and untreated cells were irradiated in the ^60^Co source (1 Gy/min, 2 min) and processed following a standard procedure previously described [58].

## Figures and Tables

**Table 1:** Cellular uptake in whole PC3 and RWPE-1 cells by PIXE. Both cells were previously incubated with AuNP-TDOTA and AuNP-BBN at 36 µg Au/mL for 4 h. The Au concentration in the whole cells is expressed in ng Au/10^6^ cells. Values are shown as the mean ± SD.

Compound	PC3	RWPE1
AuNP-TDOTA	0.33 ± 0.02	0.24 ± 0.11
AuNP-BBN	4.81 ± 0.09	1.07 ± 0.08

## Supplementary Materials

The following supporting information can be downloaded at: https://www.mdpi.com/article/10.3390/ijms23095279/s1.
