# Supplementary material for: Dose Rate Effects on the Selective Radiosensitization of Prostate Cells by GRPR-Targeted Gold Nanoparticles
Source: Int J Mol Sci. 2022 May 9;23(9):5279. doi: 10.3390/ijms23095279 (PMC9105611; doi:10.3390/ijms23095279)
Supplement: Supplementary file 1 [file ijms-23-05279-s001.zip › ijms-1709131-supplementary.pdf]

Supplementary Information

S1- Selection of the cellular dose for the irradiation studies in the presence of AuNPs

PC3 cells (without incubation with AuNPs) were exposed to a range of doses (0.5 to 6 Gy) using the PRECISA Co-60 source, described in section 4.3 of the manuscript, and dose rate of 1 Gy/min. Then, the SF (Survival Fraction) was determined by the clonogenic assay (see section 4.6 of the manuscript). As shown in Figure S1, the determined SF values, as a function of the dose, were fitted using a quadratic linear method according to [53], giving the quadratic relationship $SF = e^{-0.477D-0.007D^2}$. From its analysis, we calculated a survival fraction of 61%, 37% and 13% for 1, 2 and 4 Gy, respectively.

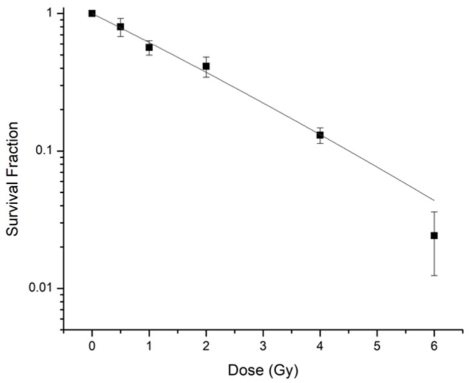

Figure S1 – Survival curve of PC3 cells after irradiation with Co-60 gamma rays, at a dose rate of 1 Gy/min. The results were calculated from independent biological replicates (n=3) and are given as the mean ± S.E.M.

S2 – Dose – rate Effect

Table S2 – Quantification of the survival fractions in PC3 cells irradiated with 2 Gy, using two different dose-rate values (25.7 mGy/min and 1 Gy/min) of Co-60, following incubation with AuNP-TDOTA and AuNP-BBN at a concentration of 36 µg Au/mL. The results were calculated from independent biological replicates (n = 2) and are given as the mean ± S.E.M.

	Control	2 Gy; 25.7 mGy/min	2 Gy; 1 Gy/min
Without AuNPs	100 ± 0.92	55.26 ± 4.26	46.61 ± 1.18
**AuNP-TDOTA**	100 ± 0.78	46.27 ± 6.42	35.22 ± 7.55
**AuNP-BBN**	100 ± 2.68	38.53 ± 2.77	2.72 ± 0.77

S3 – Quantification of the magnitude of radiosensitization: Amplification factor (AF)

The AF was calculated using equation S.1, as described elsewhere [S1]:

$$AF = \frac{SF_{without\ AuNPs} - SF_{AuNP-TDOTA/AuNP-BBN}}{SF_{without\ AuNPs}}\ x\ 100\% \qquad \text{(Equation S.1)}$$

In our study, AF refers to the radiation-induced cell death efficiency in cells incubated with AuNPs (both **AuNP-TDOTA** and **AuNP-BBN**) compared to irradiation alone (i.e. without AuNPs). As can be seen by Figure S3, the AF is higher for **AuNP-BBN** for both dose-rate values. The highest amplification for the cellular death was observed for **AuNP-BBN** when the dose-rate used was 1mGy/min.

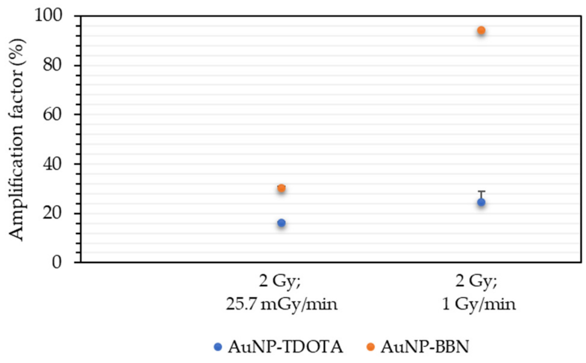

Figure S3 – Amplification factors (AF) of radiation-induced cell death efficiency in cells incubated with AuNPs (**AuNP-TDOTA** or **AuNP-BBN**) compared to irradiation alone (i.e. without AuNPs).

References

[S1] Li, S.; Penninckx, S.; Karmani, L.; Heuskin, A.C.; Watillon, K.; Marega, R.; Zola, J.; Corvaglia, V.; Genard, G.; Gallez, B., et al. *LET-dependent radiosensitization effects of gold nanoparticles for proton irradiation.* Nanotechnology, 2016, **27**: 455101.
